# Estimation of the Energy Intake Required to Prevent Body-Weight Loss in Residents of Japanese Long-Term Care Facilities

**DOI:** 10.3390/nu17142313

**Published:** 2025-07-14

**Authors:** Yuka Tachibana, Momoko Kasuya, Yuriko Haito, Masami Maeno, Kihoko Banba, Takashi Miyawaki, Naoko Komenami

**Affiliations:** 1 Department of Living Environment, Graduate School of Home Economics, Kyoto Women’s University, Kyoto 605-8501, Japan; yuka02032357@gmail.com (Y.T.); miyawakt@kyoto-wu.ac.jp (T.M.); 2 Department of Nutrition, Hyogo Prefectural Awaji Medical Center, Sumoto 656-0021, Japan; 3 Department of Food and Nutrition, Graduate School of Home Economics, Kyoto Women’s University, Kyoto 605-8501, Japan; 24131103@kyoto-wu.ac.jp; 4 Department of Nutrition, Kyoto Min-iren Asukai Hospital, Kyoto 606-8226, Japan; asukai.maeno@gmail.com; 5 Vihara Jyujo, Special Nursing Home for the Elderly, Kyoto 601-8326, Japan; k.banba@vhr10.jp

**Keywords:** elderly residents, long-term care, dietary survey, body-weight loss, energy intake

## Abstract

Background/Objectives: Proper management of food services aimed at preventing malnutrition and weight loss among residents of long-term care facilities is a critical priority. Accordingly, accurate prediction of energy intake requirements is necessary. This study aimed to estimate the energy intake required to prevent weight loss in residents of Japanese long-term care facilities. Methods: Body weight and 12-day dietary intake were measured from residents aged ≥75 years with a body mass index (BMI) < 25.0 kg/m^2^ who were consuming a regular or chopped diet. In the survey, individuals with oral intake were included, while those with swallowing problems, serious illnesses, dietary restrictions, or medications causing appetite loss were excluded. The rate of body-weight loss and the energy intake per kilogram of body weight (kcal/kg BW) during each 6-month period were calculated. The energy intake per kilogram of body weight corresponding to the rate of body-weight loss of 0% was estimated from the regression line between the rate of body-weight loss and energy intake per kilogram of body weight. Results: The data was analyzed for 99 residents (15 men and 84 women, age 89.3 ± 5.0 years, BMI 20.3 ± 2.6 kg/m^2^). From the regression results in all participants, the energy intake per kilogram of body weight corresponding to the rate of body-weight loss of 0% was 31.4 kcal/kg BW overall and 33.4 kcal/kg BW for those with a BMI < 18.5 kg/m^2^. Conclusions: The calculation of energy intake using a regression line may be able to predict the energy intake required for weight maintenance without using instrumental measurements or estimation equations, especially in the case of underweight individuals.

## 1. Introduction

The global population aged 65 years and over continues to grow due to increased global life expectancy, and Japan in particular has the highest rate of aging worldwide [1,2]. The Japanese Ministry of Health, Labour and Welfare reported that 970,000 people were using facility services as of December 2024 [3]. This has led to concerns about the additional financial burden of long-term care and medical care.

In Japanese elderly care facilities, approximately 50% of residents are at risk of malnutrition [4], with weight loss or a decrease in body mass index (BMI) known risk factors. These conditions are associated with increased morbidity and mortality, as well as functional decline [5,6]. As one of the measures to extend healthy life expectancy and reduce health inequalities, the prevention and amelioration of undernutrition among elderly individuals is a major issue [7]. Proper management of food services aimed at preventing malnutrition and weight loss among residents is a critical priority. Accordingly, accurate prediction of energy intake requirements is necessary [8,9].

Under weight-stable conditions, energy intake is expected to be equal to energy expenditure [9], which is considered to represent the energy requirement [10]. Methods for determining energy requirements include indirect calorimetry, the double-labeled water method, and predictive equations [11]. Indirect calorimetry and double-labeled water are accurate but impractical for use in facilities due to high costs and the need for trained operators [11,12,13]. Several predictive equations can estimate resting energy expenditure without requiring actual measurements, but most of these equations were not specifically developed for the elderly [14]. Furthermore, research has revealed a significant inconsistency between the resting energy expenditure measured by indirect calorimetry and the values estimated using these formulas in older hospitalized patients, with tendencies toward both overestimation and underestimation [14,15,16,17].

Body weight measurement is essential for its simplicity and ability to assess the nutritional status of residents. Therefore, it is conducted at most facilities. However, physical activity and associated illnesses can have an impact on body weight in elderly individuals. Therefore, adequacy of energy intake relative to estimated energy requirements has to be checked by measuring body weight regularly [18] and monitoring health. Weight loss suggests that energy intake may be low. Based on this background, the present study investigated the relationship between energy intake and body-weight loss among residents of long-term care facilities, excluding those requiring treatment for swallowing problems or other illnesses, with the aim of providing nutritional support and food-service management. The novelty of this study lies in its estimation of the energy intake required to prevent body-weight loss without having to experimentally measure energy expenditure in elderly residents of Japanese long-term care facilities.

## 2. Materials and Methods

### 2.1. Study Design and Recruitment

This study is a secondary analysis of existing pseudonym-processed information from a survey conducted at two long-term care facilities to assess the dietary and nutritional management of elderly individuals. The surveys were conducted during the 6 months from May to November every year from 2011 to 2016.

Informed consent was not obtained for the data used in this study because of the existing pseudonym-processed information. This study was conducted with the approval of the Research Ethics Committee of Kyoto Women’s University (approval number: 2024-45).

### 2.2. Participants

The participants were 103 residents (15 men and 88 women). The inclusion criteria for this study were age ≥ 75 years, BMI < 25.0 kg/m^2^, and intake of a regular or chopped diet. Of the two facilities, the survey was conducted for 6 years for the regular diet in Facility A, 4 years for the regular diet in Facility B, and 3 years for the chopped diet in Facility B. The survey period for each diet type varied due to the circumstances of each facility. In the survey, individuals with oral intake were included, while those with swallowing problems, serious illnesses, dietary restrictions, or medications causing appetite loss were excluded.

### 2.3. Diets

Regular diets were served to elderly residents with no masticatory difficulties. Chopped diets were prepared and served to those with impaired masticatory function by chopping the regular diet into 5 mm cubes. The need for modified food did not change for any resident during the study period.

The average dietary composition of the regular diet provided at Facility A was as follows: grains (207 g), tubers (80 g), sugar and sweeteners (7 g), nuts and seeds (1 g), green and yellow vegetables (127 g), other vegetables (199 g), fruits (32 g), mushrooms (3 g), seaweed (1 g), legumes (30 g), seafood (71 g), meat (65 g), eggs (33 g), dairy products (133 g), fats and oils (6 g), and sweets (47 g). The dietary composition of the regular and chopped diets provided at Facility B was as follows: grains (202 g), tubers (72 g), sugar and sweeteners (6 g), nuts and seeds (1 g), green and yellow vegetables (149 g), other vegetables (206 g), fruits (60 g), mushrooms (19 g), seaweed (1 g), legumes (44 g), seafood (82 g), meat (42 g), eggs (49 g), dairy products (154 g), fats and oils (8 g), and sweets (27 g).

### 2.4. Participant Characteristics

The participant characteristics—age, height (cm), body weight (kg), BMI (kg/m^2^), and care level—were confirmed at the start of the study (baseline) in May of each year from 2011 to 2016. Height was measured while standing or estimated from knee height. Body weight was measured using a standing scale or wheelchair scale, subtracting 1 kg for the weight of clothing and shoes. When using a wheelchair scale, the weight of the wheelchair and seat cushion was also subtracted. Measurements were taken mainly in the morning during times when the participants were not busy; the person who performed the measurements varied. The care level was determined based on the standards set by the Japanese Ministry of Health, Labour and Welfare, taking into consideration both physical and cognitive functions; the higher the level, the greater the need for care [19,20]. As a guideline for the need for care, care level 1 indicates that the individual is mostly independent but shows a decline in physical ability and cognitive function, requiring daily assistance, whereas care level 5 indicates that the individual is fully dependent on others for all daily activities and may be bedridden.

### 2.5. Rate of Body-Weight Loss

The rate of body-weight loss during each 6-month study period was calculated using the weight in May and November of each year. Positive values indicated weight loss, while negative values indicated weight gain.

### 2.6. Dietary Survey

A dietary survey was conducted using the food-weighing method for 3 days each in May, July, September, and November, for a total of 12 days. The survey covered breakfast, lunch, dinner, and snacks. Snacks were provided once a day between lunch and dinner to supplement the daily energy and nutrient requirements of the participants. The intake weight was calculated by subtracting the individually measured residual food weight from the provided food weight. Energy and nutrient intakes were calculated using the provided food weight and the intake weight. Energy intake per kilogram of body weight (kcal/kg BW/day) was calculated by dividing the individual participant’s average energy intake by their actual body weight in May.

### 2.7. Statistical Analysis

IBM SPSS Statistics version 28.0 (IBM Corp., Armonk, NY, USA) was used to perform the statistical analysis, and a *p*-value of <5% was considered to indicate statistical significance. In a descriptive analysis of participants’ characteristics, categorical variables are expressed as frequencies and percentages, whereas continuous variables are expressed as means with standard deviations.

One-way analysis of variance (ANOVA), followed by multiple comparisons with Bonferroni correction, was performed to examine differences in the amount of provided energy during each 6-month study period among the regular diets in Facility A and regular diets and chopped diets in Facility B.

Data on the rate of body-weight loss and energy intake per kilogram of body weight were checked for normality with the Shapiro–Wilk normality test. If the distribution was not normal, outliers were checked using box plots and excluded, and normality was assessed again. Then, Pearson’s product-rate correlation coefficient was calculated. Energy intake per kilogram of body weight corresponding to a weight-loss rate of 0% was estimated from the regression line between the rate of body-weight loss during each 6-month period and energy intake per kilogram of body weight.

Examinations by BMI group were conducted to confirm the energy intake per kilogram of body weight for weight maintenance in groups of different body sizes. Based on the undernutrition risk assessment of the Japanese Ministry of Health, Labour and Welfare [21], a BMI of 18.5–24.9 kg/m^2^ served as the reference BMI, and the participants were divided into two groups: a low BMI group (<18.5 kg/m^2^) and a normal BMI group (18.5–24.9 kg/m^2^).

## 3. Results

### 3.1. Participant Characteristics

Four participants were excluded because of outliers, leaving at total of 99 participants (15 men and 84 women) for analysis. The participants’ baseline characteristics are shown in Table 1. Mean age was 89.3 ± 5.0 years and BMI was 20.3 ± 2.6 kg/m^2^. The most common characteristic was care level 2 (light care required) (35.4%).

### 3.2. Provided Energy

The average amount of provided energy per day was 1613 ± 98 kcal/day for the regular diet in Facility A, 1629 ± 150 kcal/day for the regular diet in Facility B, and 1511 ± 176 kcal/day for the chopped diet in Facility B. One-way ANOVA showed a significant difference among the three groups (*p* = 0.007). Multiple comparisons with Bonferroni correction revealed that the chopped diet in Facility B had significantly lower provided energy compared with the regular diets in both Facilities A and B (*p* = 0.015 and *p* = 0.009, respectively). The mean provided energy per kilogram of body weight was 38 ± 5 kcal/day for the regular diet in Facility A, 34 ± 6 kcal/day for the regular diets in Facility B, and 39 ± 7 kcal/day for the chopped diet in Facility B. There was a significant difference among the three groups (*p* = 0.029). However, multiple comparisons showed no significant differences among the groups.

### 3.3. Energy and Nutrient Intake

The mean energy and nutrient intakes are shown in Table 2: 1357 ± 199 kcal/day of energy per day and 31.0 ± 5.0 kcal/kg BW/day per kilogram of body weight.

### 3.4. Relationship Between Weight Loss and Energy Intake per Kilogram of Body Weight

**Figure 1:**
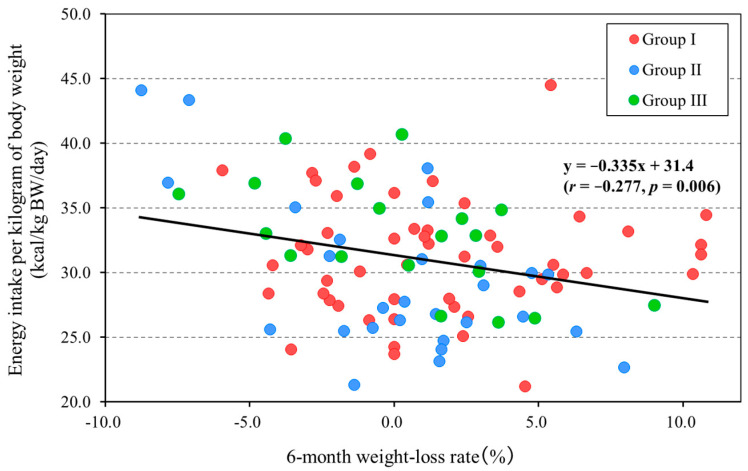
Relationship between 6-month weight-loss rate and energy intake per kilogram of body weight (All, *n* = 99). Group I, regular diet in Facility A (*n* = 52); Group II, regular diet in Facility B (*n* = 28); and Group III, chopped diet in Facility B (*n* = 19). Positive values for the 6-month weight-loss rate (%) mean weight loss and negative values mean weight gain.

Figure 1 shows a scatter plot of the rate of body-weight loss and energy intake per kilogram of body weight during 6-month periods for the overall population. The shape of the plot differs according to the diet type.

The scatter plots show that there was a significant negative correlation between the rate of body-weight loss and energy intake per kilogram of body weight (*r* = −0.277, *p* = 0.006). The equation of the regression line was y = −0.335x + 31.4 (95% confidence interval [−0.569, −0.101]), which indicates an energy intake per kilogram of body weight of 31.4 kcal/kg BW/day at a 0% rate of body-weight loss. The post hoc power analysis showed that the power (1 − β) was 0.780.

Scatter plots by BMI group are shown. The low BMI group consisted of 25 participants after 3 of the original cohort of 28 individuals were excluded as outliers. The normal BMI group consisted of 75 participants and exhibited a normal distribution. Thus, the analysis was performed with 75 participants.

**Figure 2:**
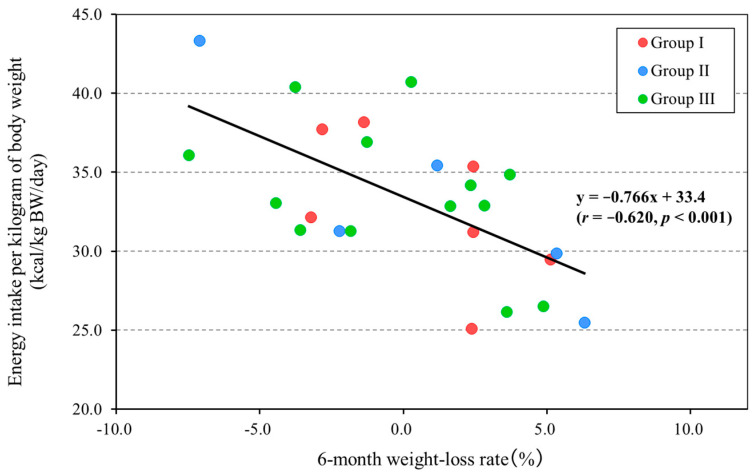
Relationship between 6-month weight-loss rate and energy intake per kilogram of body weight (BMI < 18.5 group, *n* = 25). Group I, regular diet in Facility A (*n* = 7); Group II, regular diet in Facility B (*n* = 5); and Group III, chopped diet in Facility B (*n* = 13). Positive values for the 6-month weight-loss rate (%) mean weight loss and negative values mean weight gain.

Although the low BMI group was small, there was no distributional bias regarding food type. The analysis showed a significant negative correlation between the rate of body-weight loss and energy intake per kilogram of body weight (*r* = −0.620, *p* < 0.001). The equation of the regression line was y = −0.766x + 33.4 (95% confidence interval, [−1.184, −0.348]), resulting in an energy intake per kilogram of body weight of 33.4 kcal/kg BW/day at a 0% rate of body-weight loss (Figure 2). The post hoc power analysis showed that the power (1 − β) was 0.844.

**Figure 3:**
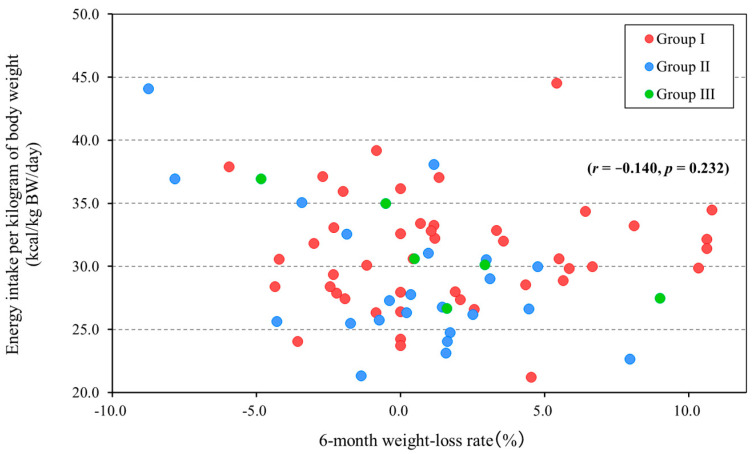
Relationship between 6-month weight-loss rate and energy intake per kilogram of body weight (BMI 18.5–24.9 group, *n* = 75). Group I, regular diet in Facility A (*n* = 45); Group II, regular diet in Facility B (*n* = 23); and Group III, chopped diet in Facility B (*n* = 7). Positive values for the 6 month weight-loss rate (%) mean weight loss and negative values mean weight gain.

Only a minority of the 75 participants with a normal BMI were on a chopped diet (7 participants). The analysis showed no significant correlation between the rate of body-weight loss and energy intake per kilogram of body weight (*r* = −0.140, *p* = 0.232) (Figure 3).

## 4. Discussion

Based on the existence of a fundamental relationship among energy intake, energy expenditure, and weight loss, this is the first study to estimate the energy intake required to prevent weight loss in elderly facility residents based on regression equations between the rate of body-weight loss and energy intake per kilogram of body weight. The results indicated that an energy intake of at least 31.4 kcal/kg/day was necessary to maintain body weight. The energy intake was 33.4 kcal/kg/day in the low BMI group, but the normal BMI group showed no association between the rate of body-weight loss and energy intake per kilogram of body weight.

The mean age of the participants in this study (89.3 years) was higher than that of the 1646 Japanese elderly facility residents in a previous study [22] conducted at the same time (85.7 years), whereas the BMI was the same in both groups (20.3 kg/m^2^). Because there were no major differences from the population in the previous study in terms of care level, the participants in the present study appear to largely exhibit similar characteristics to typical Japanese long-term facility residents.

In this study, an analysis was performed of individuals who consumed regular and chopped diets. In long-term care facilities, diets are provided in modified forms when residents are recognized as having inadequate mastication and swallowing functions. However, when the form is modified, water must be added, which decreases the energy and nutrient content per unit weight [23]. Therefore, it was difficult to estimate the energy intake required to maintain body weight by using all dietary forms for the analysis of residents. The chopped diets in this study were chopped into 5 mm cubes, and it was expected that there would be no difference in the provided energy from the regular diets. Analysis of the provided energy showed no significant difference among the regular diet at Facility A, the regular diet at Facility B, and the chopped diet at Facility B per kilogram of body weight. In addition, all diets provided more than 34.0 kcal/kg BW/day. This amount of energy satisfies the estimated energy requirements per kilogram of body weight, which is calculated according to the estimated energy requirements (physical activity level 1) for elderly individuals (aged ≥70 years) and the reference body weight (kg) in the Dietary Reference Intakes for Japanese 2015 [24]. Therefore, the diets served at the two facilities in this study were considered appropriate for the residents. The chopped diet provided approximately 100 kcal less energy compared with the regular diet, which may be because rice porridge in the chopped diet contains less energy compared with the rice in the regular diets.

A 12-week intervention trial conducted by Blundell et al. showed that fat-free mass was positively correlated with food and energy intake in obese adults. This suggests that the energy required to maintain the body’s lean tissue determines the minimal level of energy intake at meals. Furthermore, these results suggest a mechanism by which reduced fat-free mass affects appetite loss in elderly individuals with sarcopenia [25]. Therefore, we believe that the energy intake per kilogram of body weight (99 participants, 31.4 kcal/kg BW/day) at a 0% rate of body-weight change in this study may represent the minimum energy requirement to maintain weight in institutionalized elderly individuals and to thereby prevent fat-free mass. Notably, the values calculated in this study may not correspond to energy requirements based on energy expenditure. This is because the variables and measurement methods used in the calculations differ, and each has its own unique values and measurement errors [10]. Resting energy expenditure is determined mainly from indirect calorimetry, the double-labeled water method, and predictive equations [11]. In this study, we calculated the rate of body-weight loss, which depends on the balance between energy expenditure and energy intake, during 6-month periods. Energy expenditure comprises resting energy expenditure, physical activity, and diet-induced thermogenesis [26], but without measuring each of these, the energy intake required to maintain body weight was predicted by the regression line of the rate of body-weight loss and energy intake per kilogram of body weight. It is simple to multiply the predicted energy intake by body weight to calculate the energy intake required to maintain body weight, and we believe that this method is viable. For reference, Nishida et al. measured the total energy expenditure of elderly residents using the doubly labeled water method and found an energy expenditure of 25.6 kcal/kg/day for both men and women [27].

To consider the energy intake per kilogram of body weight for weight maintenance in groups of different body sizes, we divided the participants into a low BMI (< 18.5 kg/m^2^) group and a normal BMI (18.5–24.9 kg/m^2^) group, and analyzed the data. Japanese long-term care facilities have many residents with a BMI < 18.5 [22], and this was also the case in this study. Therefore, even though they had a BMI < 18.5 kg/m^2^, this study included all individuals without serious illnesses, dietary restrictions, or medications causing appetite loss. The results of the analysis revealed a significant relationship between the rate of body-weight loss and energy intake per kilogram of body weight in the low BMI group. The energy intake at a 0% rate of body-weight change was 33.4 kcal/kg BW/day for the low BMI group, which was higher than the 31.4 kcal/kg BW/day of the overall group. The reason for this may be the higher percentage of organ tissue in elderly individuals with low BMI [18]. Fat-free mass can be divided into various components with different metabolic rates, including organ tissues such as the brain, liver, heart, and kidneys, as well as skeletal muscle. The metabolic rates of these constituents influence resting energy expenditure. Organs also have a higher metabolic rate compared with muscle [28,29]. Sergi et al., in a study of body composition in elderly individuals aged ≥75 years, showed that underweight participants (BMI < 20) had a significantly lower fat mass (kg), fat mass (%), and fat-free mass (kg) compared with normal-weight participants (BMI 20–30) in both men and women but had a significantly higher fat-free mass (%) [30]. In addition, some studies have reported that, although the BMI categories differed, the resting energy expenditure was lower in the low BMI group than in the normal BMI group, while the resting energy expenditure per kilogram of body weight was higher in the low BMI group. They also indicated the importance of considering BMI categories when calculating energy expenditure [18,31]. Therefore, the relationship between the percentage of fat-free mass and organs in the elderly and energy expenditure may also be related to the regression equation for the rate of body-weight loss and energy intake per kilogram of body weight in the low BMI group in this study. In addition, weight loss in elderly individuals with low BMI suggests the influence of energy intake. However, no association was found between the rate of body-weight loss and energy intake per kilogram of body weight in the normal BMI group. Even among elderly individuals with a normal BMI, there are cases where muscle mass is low, and fat mass is high. In these cases, energy expenditure tends to be low as a result of differences in metabolic efficiency [32,33]. For this reason, it is possible that fat mass was one of the factors that affected weight loss in the normal BMI group, and this might be why there was no correlation with energy intake per kilogram of body weight.

Low BMI and weight loss are independent risk factors that affect mortality in institutionalized residents, and the presence of both factors increases mortality more than either alone [34]. Thus, elderly individuals with low BMI and weight loss need appropriate nutritional management. The present study is valuable in that it provides an objective measure of energy intake based on a total of 12 weighing surveys conducted during 6-month periods and shows a relationship with the rate of body-weight loss during the corresponding period. The predictive method and energy intake requirements for weight maintenance of this study may contribute to new nutritional support and food service management for elderly institutionalized residents.

There are several limitations to this study. First, this study did not include individuals with swallowing problems, serious illnesses, dietary restrictions, or medications causing appetite loss. Therefore, the findings might not apply to the more clinically complex populations commonly found in long-term care facilities. Second, the participants were predominantly elderly individuals aged ≥80 years, and there was a marked gender imbalance (15 men and 84 women). Therefore, the generalizability of the study results may be limited. Third, because this study had a non-interventional design, causal inferences cannot be made with certainty. The results might have been influenced by unmeasured confounders. Fourth, the decline in intestinal absorption function with aging can result in malnutrition and cachexia. However, its impact remains unclear [35,36]. Therefore, we could not clarify the impact of the decreased absorption on energy intake per kilogram of body weight and the rate of body-weight loss. Fifth, we were unable to measure body composition. As a result, it was not possible to distinguish fat mass and fat-free mass, which might have introduced bias in the relationship between energy intake per weight and body-weight loss. Although BMI is an important indicator of nutritional status in elderly individuals, it cannot reflect changes in body composition [37]. Further research should incorporate body composition analysis in order to determine how the same BMI is related to fat-free mass and skeletal muscle mass separately and the effect on energy intake.

## 5. Conclusions

The relationship between the rate of body-weight loss and the energy intake during 6-month periods in elderly residents of Japanese long-term care facilities suggests that at least 31 kcal/kg of energy per day is required to maintain body weight. Furthermore, in the population with a BMI < 18.5 kg/m^2^, the daily energy intake required to maintain body weight was 33 kcal/kg. Thus, the calculation of energy intake using a regression line may be able to predict the energy intake required for weight maintenance without using instrumental measurements or estimation equations, especially in the case of underweight individuals. However, while this study provides useful insights for nutrition planning in long-term care facilities, it has limitations in terms of the generalizability and clinical utility of the findings, as well as its inability to assess body composition. Future work should include a broader population in order to clarify whether daily energy intake is related to fat-free mass and skeletal muscle mass.

## Figures and Tables

**Table 1:** Participant characteristics (*n* = 99).

		Mean ± SD
Age (y)		89.3 ± 5.0
Height (cm)		147.8 ± 7.6
Body weight (kg)		44.3 ± 7.1
BMI (kg/m^2^)		20.3 ± 2.6
		***n* (%)**
Care level	1	9 (9.1)
	2	35 (35.4)
	3	26 (26.3)
	4	22 (22.2)
	5	7 (7.1)

BMI, body mass index. Care levels 1 to 5 were decided based on both physical and cognitive function according to the criteria established by the Japanese Ministry of Health, Labour and Welfare.

**Table 2:** Energy and nutrient intake (*n* = 99).

	Mean ± SD
Energy (kcal/day)	1357 ± 199
Protein (g/day)	50.2 ± 9.0
Fat (g/day)	36.4 ± 6.8
Carbohydrate (g/day)	200.9 ± 31.0
Salt equivalent (g/day)	6.4 ± 1.2
Protein (g/1000 kcal/day)	36.9 ± 3.1
Fat (g/1000 kcal/day)	26.8 ± 3.1
Carbohydrate (g/1000 kcal/day)	148.1 ± 8.5
Salt equivalent (g/1000 kcal/day)	4.7 ± 0.5
Energy (kcal/kg BW/day)	31.0 ± 5.0
Protein (g/kg BW/day)	1.1 ± 0.2
Fat (g/kg BW/day)	0.8 ± 0.2
Carbohydrate (g/kg BW/day)	4.6 ± 0.7

## Data Availability

Data are available from the corresponding author upon reasonable request.
